# Fevers of Alabama

**Published:** 1840-06

**Authors:** 


### FEVERS IN ALABAMA IN THE AUTUMN OF 1839.

In our first number we alluded to the extensive prevalence and mortality of the fevers of the south-west, during the past autumn, and solicited from the physicians of that region communications on the subject. We received from Dr. James C. Harris, of Jefferson, Alabama, a letter from which, although not intended for publication, we make the following extract, presenting his views of the cause and best mode of treating these fevers:

"Since the fall of 1822, when the whole valley of the Alabama was laid under disease, depopulating Cahawba, the then seat of government, so much fever has not been amongst us. Mobile, our emporium, has again been visited with yellow fever, of a most malignant type, killing, as I have been credibly informed, at least one-third of all that were attacked. At other places, the poison being less concentrated, the cases assumed a bilious congestive character, passing readily, under judicious treatment, into the remittent and intermittent forms. The ætiology of this state of things is to be found in the peculiar character of the past seasons—the luxuriance of all species of vegetation, the heat, and the protracted drought. The latter probably prevailed to a greater extent in this country, than was ever known since its early settlement, the dust lying in the roads like a bed of hot ashes, and rising in clouds almost sufficient to suffocate the traveller. The Alabama river, the Coosa, Tallapoosa, and all their tributary streams, were considerably lower than they have been known within the recollection of the oldest inhabitants. A similar combination of circumstances occurred, we are informed by *Hustis*, in his valuable work on the diseases of our State, during the summer and fall of 1822.

In the management of the cases that have occurred under my observation, the lancet, emetics, calomel, the warm bath and blisters, varied to suit the different stages of the epidemic, have been attended almost universally with success. True, I have seen in consultation, a few cases where there appeared to be a continual disposition in the system to sink; and some others where the pain in the bowels was extremely distressing, in which it was exceedingly difficult to procure proper evacuations from the liver. In the first of these conditions, sinapisms and blisters to the extremities and epigastrium, hot brandy toddy, and calomel and quinine combined, in the proportion of 10 grs. of the former to 5 grs. of the latter, repeated at intervals of two hours, and continued until three or four doses had been given, generally effected the ends desired. In the latter cases, the lancet, the warm bath, and calomel, conjoined with tart. emet. or ipecac, and aided by oil and stimulating enemas, have more certainly, and much more safely, in my opinion, accomplished all that could have been anticipated from the more active purgatives, particularly such as the croton oil, gamboge, scammony, &c. The relaxing and powerfully remedial effects of the lancet, under such circumstances, can only be fully appreciated by those who have witnessed its beneficial results; and although I have not ventured on its employment more than twice during the actual chill, still I have repeatedly employed it in cases of deep congestion, where the extremities and surface were cool, and the prostration sudden and apparent, and in every instance, so far as I now recollect, with good effect. So thoroughly am I impressed with the importance of the lancet, in all febrile affections, that I have been led sometimes almost to conclude, that were it practised now, as in the days of ***Rush***, the size of all our purgative remedies might be very considerably reduced, and as beneficial results might be obtained from 20 grs. of calomel, combined with a small proportion of tartar emetic or ipecac, as from 50 or 100 grs. of the former article, unaided and uncombined—and that, too, with much less danger of doing permanent injury to the system. But where copious depletion has been neglected in the forming stages of our fevers, and dangerous and extensive determinations take place, large doses of calomel may be employed with safety and advantage. This subject occupied my thoughts at an early period of my professional career, and the above is stated as the result of my experience, in public and private practice for seven or eight years." Y.

November, 1839.

